# Computational Insights into The Neuroprotective Action of Riluzole on 3-Acetylpyridine-Induced Ataxia in Rats

**Published:** 2013-07-02

**Authors:** Samira Abbasi, Mehdi Edrisi, Amin Mahnam, Mahyar Janahmadi

**Affiliations:** 1 Department of Biomedical Engineering, School of Engineering, University of Isfahan, Isfahan, Iran; 2 Neuroscience Research Center and Department of Physiology, Faculty of Medicine, Shahid Beheshti University (Medical Campus), Tehran, Iran

**Keywords:** Ataxia, Riluzole, Potassium Channels, Neuroprotection, Computer Simulation

## Abstract

**Objective:**

Intra-peritoneal administration of riluzole has been shown to preserve the membrane properties and firing characteristics of Purkinje neurons in a rat model of cerebellar ataxia induced by 3-acetylpyridine (3-AP). However, the exact mechanism(s) by which riluzole restores the normal electrophysiological properties of Purkinje neurons is not completely understood. Changes in the conductance of several ion channels, including the BK channels, have been proposed as a neuro protective target of riluzole. In this study, the possible cellular effects of riluzole on Purkinje cells from 3-AP-induced ataxic rats that could be responsible for its neuro protective action have been investigated by computer simulations.

**Materials and Methods:**

This is a computational stimulation study. The simulation environment enabled a change in the properties of the specific ion channels as the possible mechanism of action of riluzole. This allowed us to study the resulted changes in the firing activity of Purkinje cells without concerns about its other effects and interfering parameters in the experiments. Simulations were performed in the NEURON environment (Version 7.1) in a time step of 25 μs; analyses were conducted using MATLAB r2010a (The Mathworks). Data were given as mean ± SEM. Statistical analyses were performed by the student’s t test, and differences were considered significant if p< 0.05.

**Results:**

The computational findings demonstrated that modulation of an individual ion channel current, as suggested by previous experimental studies, should not be considered as the only possible target for the neuro protective effects of riluzole to restore the normal firing activity of Purkinje cells from ataxic rats.

**Conclusion:**

Changes in the conductance of several potassium channels, including voltage- gated potassium (Kv1, Kv4) and big Ca^2+^-activated K^+^ (BK) channels may be responsible for the neuro protective effect of riluzole against 3-AP induced alterations in the firing properties of Purkinje cells in a rat model of ataxia.

## Introduction

The electrophysiological properties of Purkinje cells play an important role in the normal function of the cerebellum, including fine-tuning movements, posture, coordination, and timing of motor behaviors (1-4). Cerebellar ataxia, a disease characterized by disturbance in coordination, postural instability, gait abnormalities, and intention tremor is the result of changes in the physiological function of cerebellar Purkinje cells (1, 3, 4). Currently neuro protective agents are promising therapies for treatment of neurodegenerative diseases, such as cerebellar ataxia. Several experimental studies on animal models of ataxia (5-8) have demonstrated a significant neuro protective effect for riluzole in ataxia. Experimental studies have shown that riluzole restores the normal firing activity in ataxic Purkinje cells (1, 4). It is believed that the therapeutic effect of riluzole may be through its significant effect on the electrophysiological properties of Purkinje cells (1, 4, 9). However, the exact mechanism involved in neuro protection by riluzole is unclear

In study by Janahmadi et al. on a rat model of cerebellar ataxia, behavioral and electrophysiological methods were used to explore the therapeutic potential of riluzole (1). According to their results, *in vivo* treatment with riluzole almost completely inhibited neuronal degeneration in the cerebellar Purkinje cell layer and partially prevented the development of ataxia. These researchers reported that the firing patterns of Purkinje cells changed from a regular pattern in the control group to an irregular pattern in ataxic rats. Janahmadi et al. indicated that riluzole treatment caused increased firing frequency of Purkinje cells obtained from ataxic rats. Riluzole preserved the membrane properties and firing characteristics of Purkinje neurons and restored the electrophysiological characteristic of Purkinje cells, such as the amplitude of after-hyper polarization potential (AHP), spike duration and amplitude of action potentials (AP) compared to control conditions (1, 4). The author suggested that neuro protective effects of riluzole against 3-acetylpyridine (3-AP) toxicity could be related to the enhancement of big Ca^2+^-activated K^+^ (BK) channel activity (1) or modulation of voltage-gated potassium (Kv1) channels (4).

In several experimental studies on other cells, riluzole has been reported to activate several types of K^+^ channels (10-13), however it blocks Kv4.3 currents (14, 15). Experimental studies have also shown that riluzole can inhibit voltage gated Na^+^ channels (10, 16, 17). While spontaneous discharge activity is reduced in ataxic Purkinje cells, Goudarzi et al. (9) have suggested that enhancement of spontaneous discharges in ataxic Purkinje cells by riluzole is due to the inhibition of fast inactivating potassium channels (Kv4). Alviña and Khodakhah (14) have proposed that a suitable therapeutic target for the treatment of type-2 episodic ataxia might be the Ca^2+^-dependent K^+^ channel in Purkinje cells.

Computational models of neurons are important tools for investigating different aspects of their complex behavior. In the simulation environment it is possible to investigate how each specific ionic current can affect the neurons’ electrophysiological properties. This is an excellent method for mimicking cell response in the presence of channel blockers without concern for blocker side-effects or numerous other uncontrollable parameters that may influence the results in an experimental study.

Therefore, in this study, we simulated the electrical behaviors of Purkinje cells to determine the possible mechanisms of action of riluzole on their firing behavior and electrophysiological properties. Based on experimental evidences suggested in previous studies, the maximum conductance of different ion channels were changed as the possible mechanism of action of riluzole and its effects on the firing activity of the Purkinje cell were studied. We tested hypotheses of the effects of riluzole on different ion channels in a simulation environment by investigating whether described changes produced firing activities similar to that experimentally recorded from Purkinje cells of ataxic rats treated by riluzole.

## Materials and Methods

### Computer simulations

In this computational stimulation study, we used models of normal and ataxic Purkinje cells to study the possible cellular basis of altered firing behavior of Purkinje neurons in a rat model of ataxia. We also studied the cellular mechanisms of neuroprotection by riluzole. The behavior of normal Purkinje cells with tonic firing was used as a preliminary reference, while the main references for the firing activity of normal, ataxic and riluzole treated cells were experimental recordings that previously published by Janahmadi et al. (1).

The basic computational model of Purkinje cells provided by Akemann and Knopfel (18) was used to simulate the tonic firing activity of normal Purkinje cells. The model is a slightly modified version of the model provided by Khaliq et al. (19) for normal cells. Only the soma is included in this model which consists of eight types of ion channels (resurgent Na^+^, non-resurgent Na^+^, Ih, Kv1, Kv3, Kv4, BK, P-type Ca^2+^) and leak channel. Simulations with the original model qualitatively mimicked the experimental recordings from normal cells. However, to match the frequency of firing and input impedance, the maximum conductance of the resurgent Na^+^ current was slightly increased from 16 mS/cm^2^ to 16.5 mS/cm^2^; the diameter and length of soma were both increased from 20 μm to 30 μm.

To simulate an ataxic Purkinje cell, we used a modified version of the ataxic Purkinje cell model provided by Khaliq et al. (19). The size of the ataxic Purkinje cells has been reported to be smaller than normal cells and their input resistance is higher (1, 4). To match the input resistance of the model with the experimental data, the diameter and length of the modeled Purkinje cell were both reduced from 30 μm to 20 μm. A change in cell size does not significantly affect firing activity of the model cell.

Simulations were performed in the NEURON environment (Version 7.1). The simulations were run with a time step of 25 μs. Analyses were conducted using MATLAB r2010a (The Math works). Statistical analyses were performed by the student’s t test and differences were considered significant if p< 0.05.

### Electrophysiological assessment

We assessed electrophysiological characteristics of the simulated cells during three intervals of two minutes of the simulations. The specifications of the repetitive APs were expressed as mean ± SEM. The electrophysiological characteristics that compared the firing activity of the simulated cells with the experimental recordings were: firing frequency of the cell, amplitude of the APs, duration of the APs, amplitude of the AHP and input resistance (1). The amplitude of the APs was measured from the baseline to the peak. Action potential duration was calculated as the duration measured at the half amplitude. Action potential AHP was measured from the baseline to the negative peak of the AP. The input resistance was calculated from the change in steady-state voltage evoked by the injection of hyperpolarizing current steps (-0.07 to 0.1 nA at 0.01 nA increments) from a resting potential of -60 mV

## Results

### Comparison of the electrophysiological characteristics of control and ataxic Purkinje neurons

**Fig 1:**
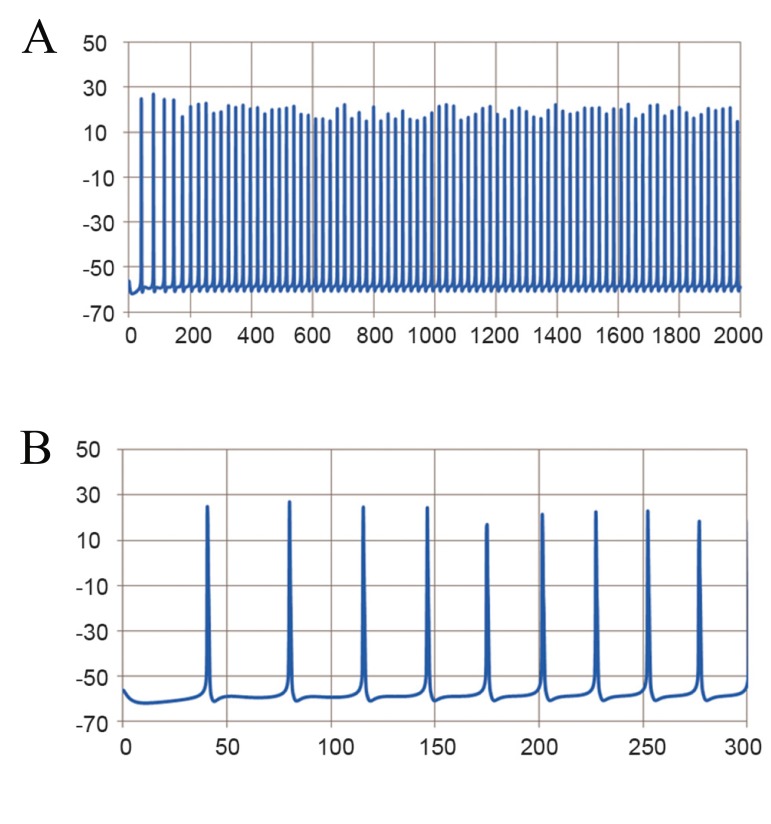
Simulated spontaneous tonic firing of the Purkinje cell under normal conditions. A. Fast time scale and B. Slow time scale.

Simulated normal Purkinje cell somata exhibited spontaneous tonic firing (Fig 1) at 41 ± 2 Hz while sitting at an average membrane potential of -56 mV. Regularly spaced APs had a mean amplitude of 77.56 ± 5.65 mV and duration of 0.7 ± 0.1 ms. The AHP amplitude was - 4.72 ± 0.26 mV and the average input resistance was 118.84 ± 5 MΩ.

**Fig 2:**
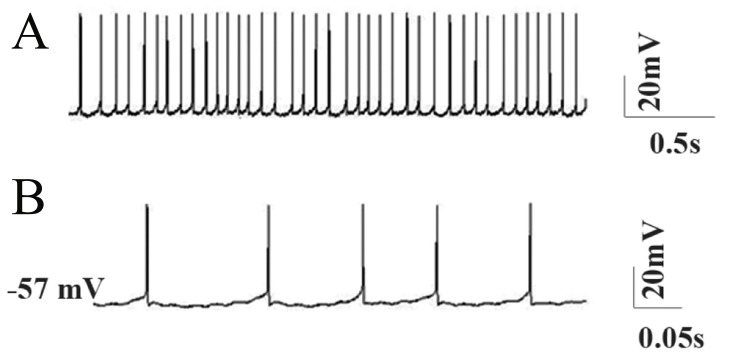
Spontaneous tonic firing pattern of Purkinje neurons under whole cell current clamp. A. Fast time scale and B.Slow time scale.

Despite slight differences, these results were consistent with the experimental data (1, 4) recorded from Purkinje cells in the presence of synaptic blockers (Fig 2). Experimental data showed that Purkinje cells had a mean input resistance of 111.76 ± 7.3 MΩ and fired spontaneously at 39.38 ± 5.83 Hz during whole cell current clamp recording from a mean resting membrane potential of -57.8 ± 2.43 mV. The regularly spaced APs had a mean amplitude of 50 ± 2.8 mV and mean duration of 0.5 ± 0.01 ms (1).

**Fig 3:**
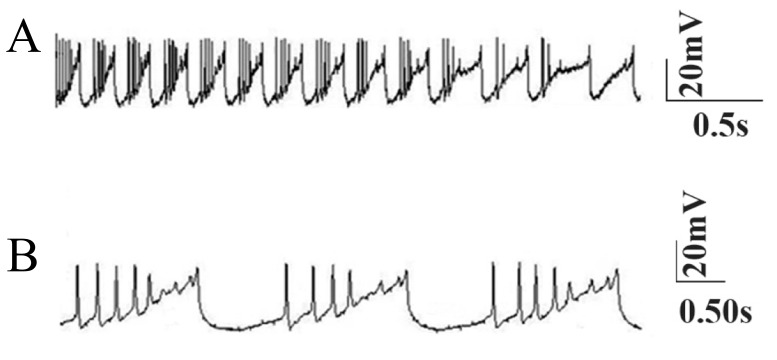
Bursting activity of a real ataxic Purkinje neuron. A. Fast time scale and B.Slow time scale.

Janahmadi et al. (1) recorded the firing activity of ataxic Purkinje cells obtained from ataxic rats treated with the neurotoxin, 3-AP. Purkinje cells from these rats had considerably higher input resistance (166.9 ± 6.14 MΩ). The overall discharge activity was significantly lower (10 ± 1.9 Hz), but the resting membrane potential of Purkinje cells remained almost unchanged (-57.06 ± 1.5 mV). The tonic repetitive firing pattern of Purkinje cells changed into a bursting mode following 3-AP treatment (Fig 3).

There was a significantly smaller AHP amplitude observed in the Purkinje cells obtained from 3-AP treated rats (-5 ± 1.3 mV) compared to normal Purkinje cells (-7.41 ± 0.4 mV). Treatment with 3-AP was also associated with a significant decrease in amplitude observed in ataxic rats (45 ± 2 mV) compared with normal rats (50 ± 2.8 mV). An increase in the duration of APs was noted in ataxic rats (0.9 ± 0.1 ms) compared with normal rats (0.5 ± 0.01 ms) (1, 4).

The modified Purkinje cell model for ataxic condition can mimic the changes observed in an experimental situation. The average input resistance was 180.75 ± 9.06 MΩ. We recorded a spontaneously tonic firing of 20 ± 1 Hz; the AHP amplitude was -3.6 ± 0.9 mV

**Fig 4:**
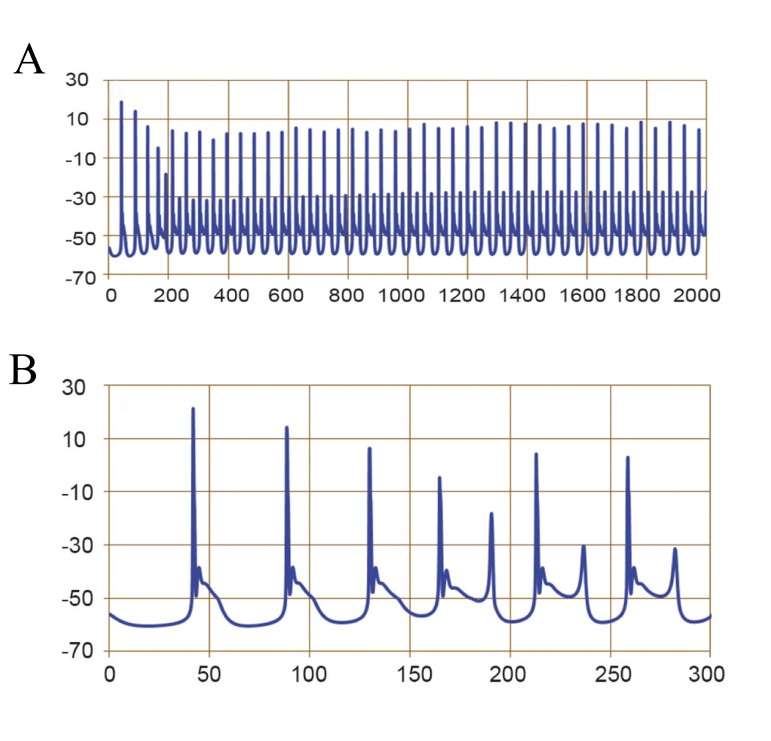
Simulated spontaneous firing of the Purkinje cell under ataxia conditions. A.Fast time scale and B.Slow time scale.

The mean amplitude of APs was 62.5 ± 11.9 mV, with a duration of 1 ± 0.1 ms (Fig 4). The resting membrane potential of the Purkinje cells remained unchanged (-56 mV).

Electrophysiological characteristics of real and simulated cells in control and ataxic conditions are summarized in table 1. The changes in these parameters from simulated normal to ataxic cells confirmed those obtained from experimental data. The results agreed with simulations reported by Akemann and Knopfel (18) and Khaliq et al. (19).

**Table 1:** Electrophysiological characteristics of real and simulated Purkinje cells under normal and ataxia conditions


Electrophysiological parameters	Normal purkinje cell	Purkinje cells from ataxic rats	Simulated normal purkinje cell	Simulated ataxic purkinje cell

**Frequency (Hz)**	39.38 ± 5.83	10 ± 1.9	41 ± 2	20 ± 1
**AP amplitude (mV)**	50 ± 2.8	45 ± 2	77.56 ± 5.65	62.5 ± 11.9
**AHP amplitude (mV)**	-7.41 ± 0.4	-5 ± 1.3	-4.72 ± 0.26	-3.6 ± 0.9
**AP duration (ms) **	0.5 ± 0.01	0.9 ± 0.1	0.7 ± 0.1	1 ± 0.1
**Input resistance (MΩ)**	111.76 ± 7.3	166.9 ± 6.14	118.84 ± 5	180.75 ± 9.06


### Neuroprotective effects of riluzole on ataxic Purkinje neurons

**Fig 5:**
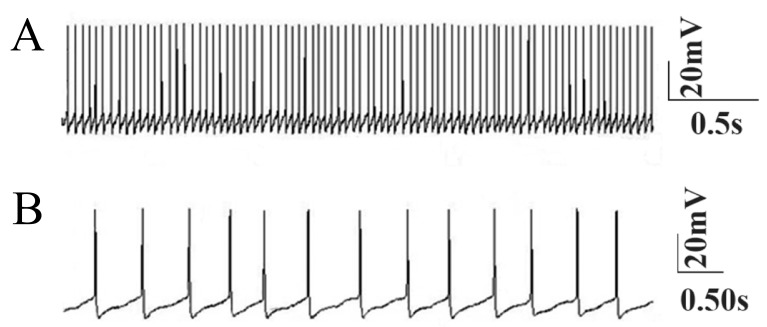
The spontaneous firing pattern of Purkinje neuron recorded from 3-AP + riluzole treated rats. A. Fast time scale and B. Slow time scale.

Janahmadi et al. (1) reported that most Purkinje neurons from 3-AP + riluzole treated rats exhibited regular tonic firing of Na^+^ spikes (Fig 5). The mean input resistance of these cells almost returned to normal conditions (117 ± 3.35 MΩ). Treatment with 3-AP + riluzole did not produce significant change in resting membrane potential of Purkinje neurons, but increased the mean firing frequency (25 ± 3 Hz) compared to ataxic neurons (10 ± 1.94 Hz). In these cells, the amplitude and duration of APs returned to the control values. Riluzole also significantly increased the amplitude of AHP compared to ataxic neurons (1, 4).

In order to explore how riluzole affects the firing activity of Purkinje cells, we examined the effects of riluzole in an ataxic cell model as changes in different ionic currents and compared the resultant firing activities of Purkinje cells with normal and ataxic cells.

Evidence from experimental data suggested that riluzole can activate several types of K^+^ channels including BK and Kv1 (10-13), but blocks the Kv4.3 current (14, 15). It may also inhibit voltage gated Na^+^ channels (10, 16, 17). Based on these data, our computational experiments focused on changes in these ionic currents as possible mechanisms of action of riluzole.

The results of the simulations indicated that inhibition of voltage gated Na^+^ channels in ataxic Purkinje cells suppressed cell firing but did not change the firing activity of the cell towards normal tonic activity. Therefore, in the present theoretical study we considered activation of the BK, Kv1 and Ih channels and inhibition of Kv4 channels to be the sole mechanisms underlying the neuroprotective effect exerted by riluzole; thus, these channels were extensively examined.

In the model we changed conductance of individual channel types in steps of 10% of the original value and evaluated changes in the firing activity of the ataxic Purkinje cell model.

**Fig 6:**
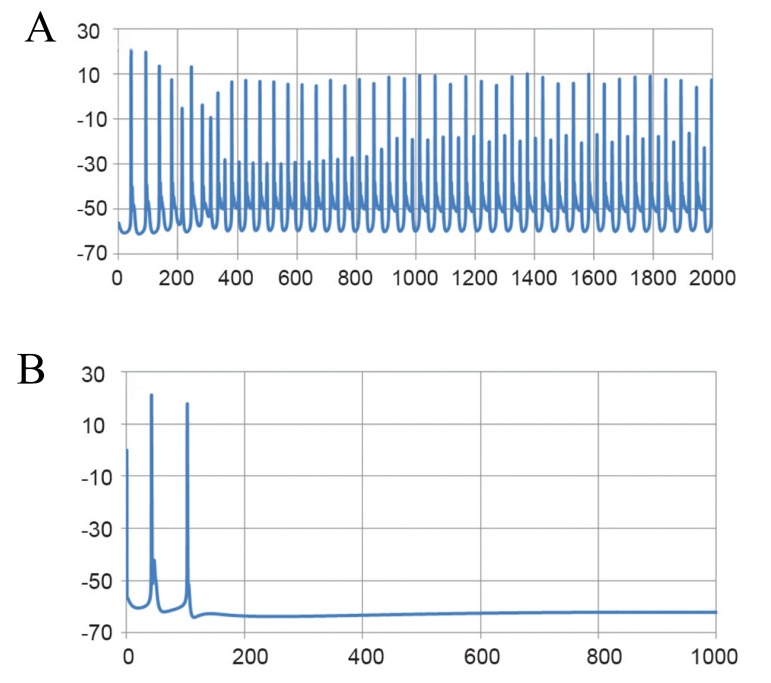
Spontaneous firing of an ataxic Purkinje neuron with increased BK channel conductance. A. 30% increase and B. 40% increase.

As shown in figure 6A, a 30% increase in the conductance of BK channels in ataxic Purkinje cells did not significantly affect neuronal response. Additional increases (≥40%) in BK channel conductance suppressed cell firing and the membrane potential rested at -62.3 mV (Fig 6B).

**Fig 7:**
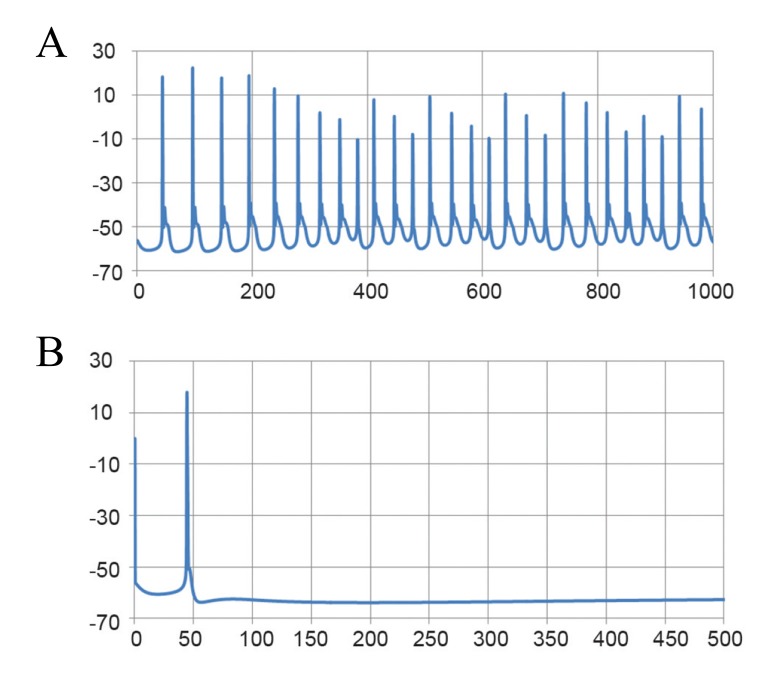
Spontaneous firing of an ataxic Purkinje neuron with increased Kv1 channel conductance. A.40% increase and B.50% increase.

Increasing the conductance of the Kv1 channels enhanced the regularity of the firing activity as seen in figure 7A at the 40% increase, however it could not be restored to the normal condition before the cell’s firing activity was suppressed (Fig 7B).

**Fig 8:**
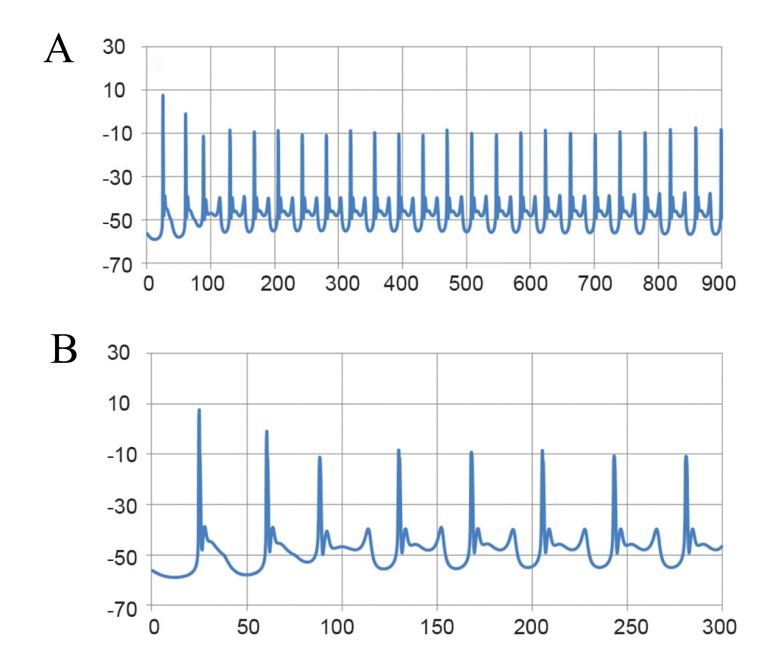
Spontaneous firing of a simulated ataxic Purkinje neuron with a 50% increase in Ih channel conductance. A. Fast time scale and B.Flow time scale.

As shown in figure 8, increased conductance of the Ih channels by 50% decreased the AHP amplitude by approximately 80% and APs amplitude by approximately 20%. However, these changes could not also restore the normal firing activity of the Purkinje cells

**Fig 9:**
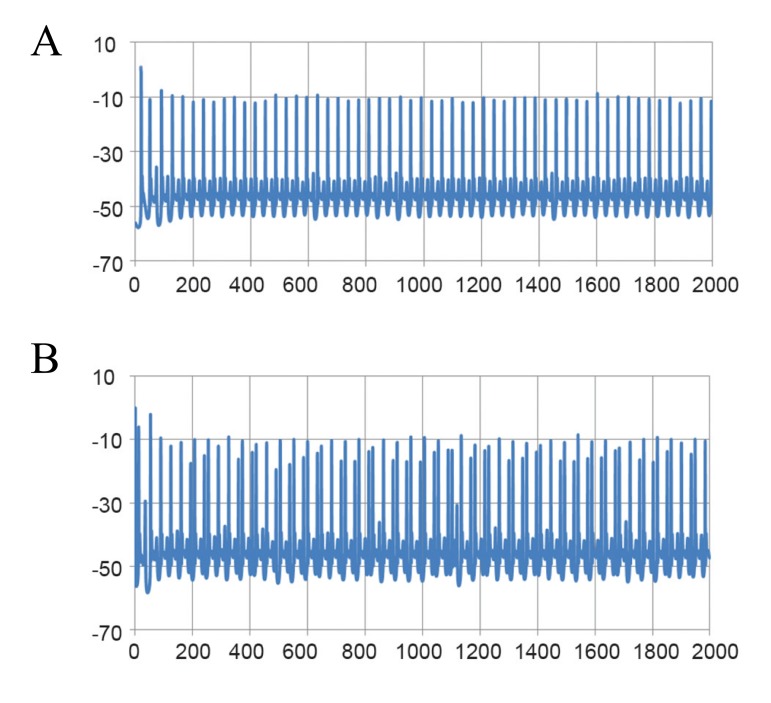
Spontaneous firing of a simulated ataxic Purkinje neuron with reduction in the conductance of Kv4 channels. A. 10% reduction and B. 20% reduction.

Inhibition of Kv4 channels increased the firing rate of the ataxic cell model and decreased the AHP and APs amplitudes. Figures 9A and B show the firing activity of simulated Purkinje cells following 10% and 20% reduction in the Kv4 channel conductance, respectively.

The simulations showed that changes in the conductance of individual channels alone could not mimic the neuro protective effect of riluzole as observed in the experimental situations. Therefore, it could theoretically be suggested that changes in several ionic conductances might be involved in neuro-protection mediated by riluzole.

**Fig 10:**
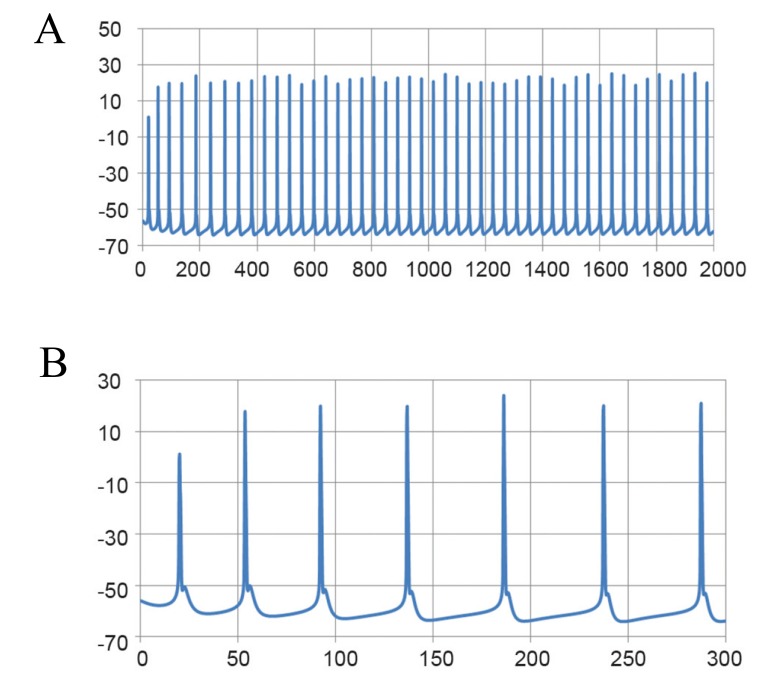
Spontaneous firing of a modeled ataxic Purkinje neuron with a 10% reduction in Kv4 channel conductance along with 75% enhancement in BK channel conductance. A. Fast time scale and B. Slow time scale.

As shown in figure 10, a 10% reduction in the conductance of Kv4 channels along with a 75% increase in the conductance of BK channels mimicked the neuro-protective effect of riluzole on ataxic Purkinje cells and restored the firing activity of the ataxic Purkinje cell model to the control level. The firing pattern of the Purkinje cell became regular and exhibited spontaneous tonic firing at 24 ± 2 Hz. Amplitudes of the APs restored to normal conditions (79.6 ± 2 mV) and the AHP amplitude increased to -7.6 ± 0.2 mV. A 10% decrease in the conductance of Kv4 channels and 75% increase in the conductance of BK channels were the minimum amount of change required to restore normal firing of the Purkinje cell model. Changes greater than these minimum values resulted in slight quantitative changes in the response, at least for a range of values, and quantitatively produced responses closer to the control neurons.

To restore input resistance to the normal condition, the size of modeled Purkinje cell was altered. This finding was consistent with experimental results that reported the size of Purkinje cells treated with riluzole as approximately that of normal Purkinje cells (1, 4). By restoring the size of modeled ataxic cell to that of normal conditions, the average input resistance was restored to 123.27 ± 7.25 MΩ.

**Fig 11:**
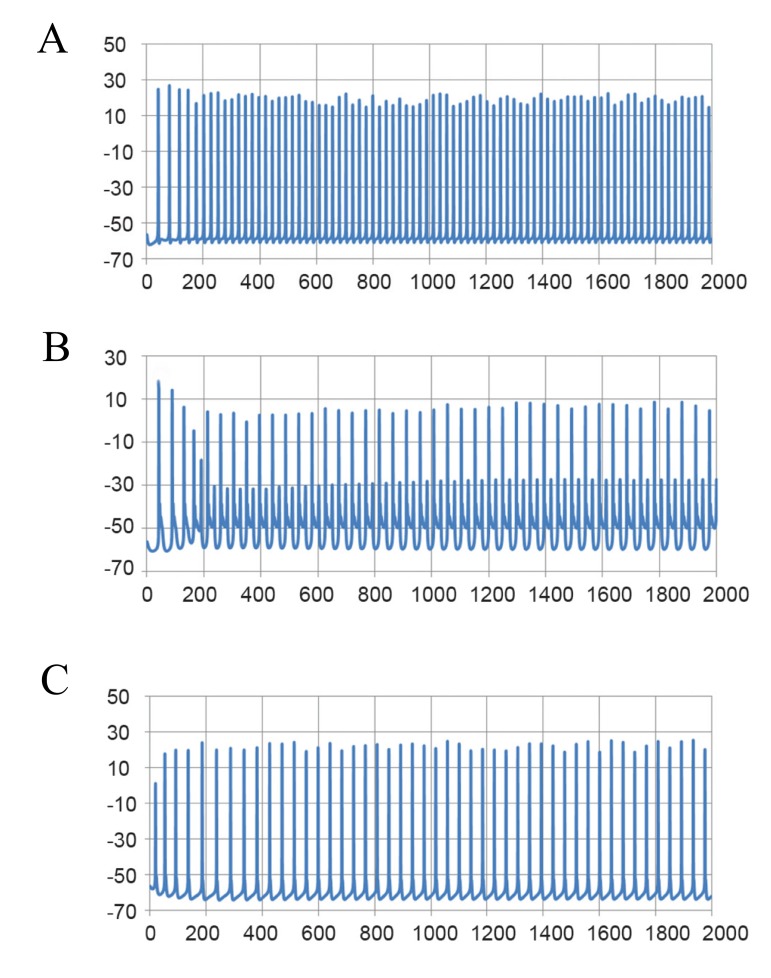
Simulated spontaneous tonic firing of the Purkinje cell in A. Normal, B. Ataxia, and C. Riluzole-treated ataxia conditions.

The simulated responses of Purkinje cells for normal, ataxic and riluzole treated ataxic conditions are shown in figure 11.

Simultaneous change in BK and Kv4 conductances were not the only possible alterations in the electrophysiological properties of the ataxic Purkinje cells that could restore their firing activity. Another possible mechanism for the riluzoleinduced neuro protection against 3-AP toxicity could be through a combination of changes in the conductances of Kv4 and Kv1 channels, so that a 10% decrease in the conductance of Kv4 channels could also simulate the neuro protective effect of riluzole on the modeled Purkinje cells when accompanied by a 90% increase in the conductance of Kv1 channels. A similar result was also achieved by 80 and 40% increases in the conductance of Kv1 and Ih channels, respectively. In addition, a minimum increase of 70 (BK channel) and 40% (Ih channel) restored the firing activity of the modeled ataxic Purkinje cell to the normal condition.

However, changes in the conductance of Kv4 and Ih channels did not restore the electrophysiological properties of the modeled ataxic Purkinje cell to the normal condition.

There are also several, three channel combinations of changes in conductance which have resulted in restoring firing activity of the model Purkinje cell. Table 2 summaries these combinations.

**Table 2:** Summary of the results, which demonstrate the minimum changes in the conductance of different channels which may restore the firing activity of ataxic Purkinje cells to normal


Channel modulation	Ability to restore electrophysiological characteristics of ataxia to normal conditions

**Kv4 inhibition**	No
**BK stimulation **	No
**Kv1 stimulation **	No
**Ih stimulation **	No
**Kv4 inhibition (10%) and BK stimulation (75%) **	Yes
**Kv4 inhibition (10%) and Kv1 stimulation (90%) **	Yes
**Kv4 (10%) inhibition, Kv1 (40%) and BK (40%) stimulation**	Yes
**Ih (40%) and BK (80%) stimulation **	Yes
**Ih (40%) and Kv1 (80%) stimulation **	Yes
**Ih (40%), Kv1 (40%) and BK (40%) stimulation **	Yes


## Discussion

In the present study we discussed the possible cellular mechanisms of action of riluzole as a neuro protective agent. A simulation environment was used to evaluate the contributions of different ion channels previously proposed (1, 9-17) to determine if changes in their conductances could explain restoration of the electrophysiological properties of these cells.

The simulation findings suggested that modulation of the conductance of each of the proposed channels alone could not be responsible for the electrophysiological effects of riluzole on ataxic cells. However, several combinational effects on two or three of the proposed channels simulated the observed neuroprotective effects of riluzole on the firing activity of Purkinje cells. These proposed combinations consisted of: Kv4 inhibition and BK activation; Kv4 inhibition and Kv1 activation; Kv4 inhibition with Kv1 and BK activation; Ih and BK activation; Ih and Kv1 activation; or Ih, Kv1 and BK activation.

Inhibition of Kv4 along with activation of Kv1 and BK in different cells treated by riluzole were reported in several previous studies (1, 9-17). However no experimental evidence has been found in the literature for an increase in Ih channel current in the presence of riluzole. Therefore, it is most possible that a combination of several ionic channels, including Kv1, Kv4 and BK, are responsible for the neuro protective effects of riluzole.

**Fig 12:**
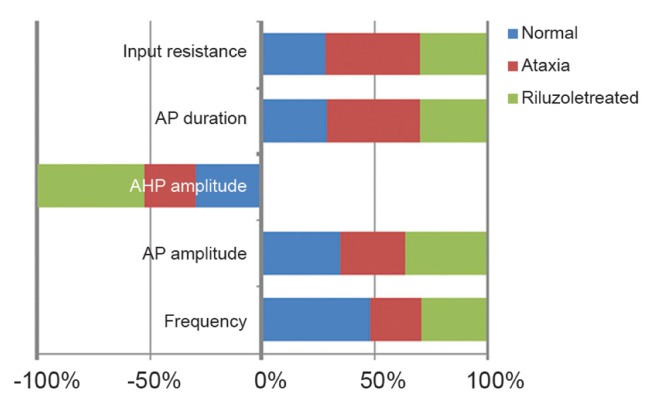
Simulated effects of riluzole on the electrophysiological characteristics of Purkinje neurons, including A. Input resistance, B. Action potential (AP) duration, C. AHP amplitude, D.AP amplitude and E: Frequency.

**Fig 13:**
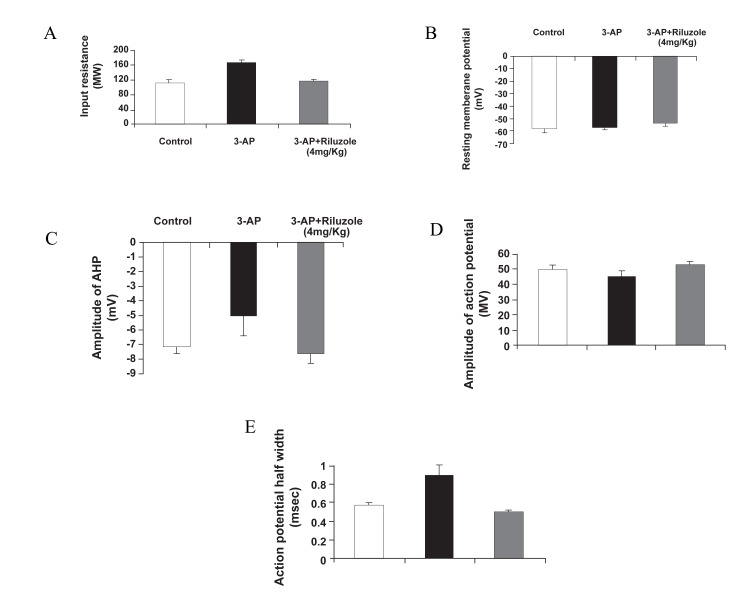
Real effects of riluzole on the electrophysiological characteristics of Purkinje neurons, including A.Input resistance, B.Resting membrane potential, C.AHP amplitude, D. Action potential (AP) amplitude and E. AP half width (1).

Figures 12 and 13 summarize the simulated and real effect of riluzole on the electrophysiological characteristics of Purkinje neurons, respectively.

In comparison with control and riluzole cotreatment conditions the input resistance of PCs signiﬁcantly improved. Both resting membrane potentials of PCs remained unchanged, however there was a significant decrease in the APs amplitude and a significant increase in its duration in ataxic rats. Both parameters almost returned to control values in the riluzole co-treated groups. The results were almost the same in the real and simulated effects of riluzole on the electrophysiological characteristics of Purkinje neurons.

The results of this study may be used in future experiments to determine more exactly the mechanism(s) by which riluzole restores the electrophysiological properties of neuronal cells against normal conditions.

## Conclusion

Simulation results indicated that changes in the conductance of individual channels alone such as the increment of BK channels (1) or Kv1 channels conductance (4) and inhibition of Kv4.3 currents (14, 15) could not reproduce the neuroprotective effect of riluzole as observed in the experimental studies. Therefore, it seemed that changes in the conductance of several potassium channels, including Kv1, Kv4 and BK, might be responsible for the neuroprotective effect of riluzole against 3-AP induced alterations in the electrophysiological characteristics of Purkinje neurons in a rat model of ataxia.
